# Demonstration of nanoimprinted hyperlens array for high-throughput sub-diffraction imaging

**DOI:** 10.1038/srep46314

**Published:** 2017-04-10

**Authors:** Minsueop Byun, Dasol Lee, Minkyung Kim, Yangdoo Kim, Kwan Kim, Jong G. Ok, Junsuk Rho, Heon Lee

**Affiliations:** 1 Department of Materials Science and Engineering, Korea University, Seoul 02842, Republic of Korea; 2 Department of Mechanical Engineering, Pohang University of Science and Technology (POSTECH), Pohang 36763, Republic of Korea; 3 Department of Mechanical and Automotive Engineering, Seoul National University of Science and Technology, Seoul 01811, Republic of Korea; 4 Department of Chemical Engineering, Pohang University of Science and Technology (POSTECH), Pohang 36763, Republic of Korea; 5 National Institute of Nanomaterials Technology (NINT), Pohang 37676, Republic of Korea

## Abstract

Overcoming the resolution limit of conventional optics is regarded as the most important issue in optical imaging science and technology. Although hyperlenses, super-resolution imaging devices based on highly anisotropic dispersion relations that allow the access of high-wavevector components, have recently achieved far-field sub-diffraction imaging in real-time, the previously demonstrated devices have suffered from the extreme difficulties of both the fabrication process and the non-artificial objects placement. This results in restrictions on the practical applications of the hyperlens devices. While implementing large-scale hyperlens arrays in conventional microscopy is desirable to solve such issues, it has not been feasible to fabricate such large-scale hyperlens array with the previously used nanofabrication methods. Here, we suggest a scalable and reliable fabrication process of a large-scale hyperlens device based on direct pattern transfer techniques. We fabricate a 5 cm × 5 cm size hyperlenses array and experimentally demonstrate that it can resolve sub-diffraction features down to 160 nm under 410 nm wavelength visible light. The array-based hyperlens device will provide a simple solution for much more practical far-field and real-time super-resolution imaging which can be widely used in optics, biology, medical science, nanotechnology and other closely related interdisciplinary fields.

The resolution of conventional optical systems is constrained by diffraction limit^1^ which prevents imaging of sub-wavelength features. As a result, a variety of imaging techniques have been reported to overcome diffraction limit. Near-field scanning optical microscopy has been proposed to offer super-resolution imaging by collecting the near-field information using a sharp tip in contact with the object^2^, followed by other fluorescence imaging methods such as, stochastic optical reconstruction microscopy (STORM)^3^, photo activated localization microscopy (PALM)^4^, stimulated emission depletion microscopy (STED)^5^ and structured illumination microscopy (SIM)^6^. A new concept of superlens has received scientific attention since the first proposal of the perfect lens^7^ based on negative refractive index, for its ability to restore evanescent waves and thereby, achieve super-resolution imaging. Early experimental demonstrations of the superlensing effect are limited to near-sight^8^,^9^,^10^, although the possibility of superlensing in the far-field^11^,^12^ with reasonable resolution has also been introduced. Around the same time, it has also been demonstrated that hyperlenses^13^,^14^,^15^,^16^,^17^,^18^,^19^,^20^,^21^,^22^,^23^,^24^,^25^,^26^ extend super-resolution imaging to the far-field thanks to their hyperbolic dispersion profiles.

Though hyperlenses have proved that sub-diffractional imaging in real-time at UV and visible frequencies range is possible, they have been neither used in real imaging applications such as biomolecular imaging, nor yet commercialized. While the previously demonstrated hyperlens systems focus on a single hyperlens, which have small observation area and thus require precise positioning of non-artificial objects such as live cells, an array type of hyperlens has been considered as a prominent solution for the practical applications. However, it is extremely difficult to fabricate a perfect array of a spherical-pattern substrate for hyperlens devices using either the focused ion beam (FIB) process or electron beam lithography (EBL), which have been frequently used to make the previous hyperlenses and are not suitable for such a large-scale hyperlens device due to low productivity and high cost.

In this paper, we use nanoimprint lithography (NIL)^27^,^28^ to solve the limitation of previous hyperlens fabrication processes. Based on a simple pattern transfer process, the NIL technique is able to easily replicate the shape of a master stamp and make a perfect array of hemispheres over a large area with very low cost. Finally, we fabricate a perfect large-scale hyperlens device on a replicated hexagonal array of hemisphere (HAHS) substrate directly printed and pattern-transferred from the master mold, followed by metal-dielectric multilayer deposition by electron beam evaporation. With a hyperlens integrated simple wide-field microscope setup, we experimentally confirm that sub-diffractional objects with a separation of 160 nm, which is much smaller than diffraction limit of the given optical system of 280 nm, are clearly resolved.

## Theory and Methods

### Principles of hyperlens and numerical studies

**Figure 1:**
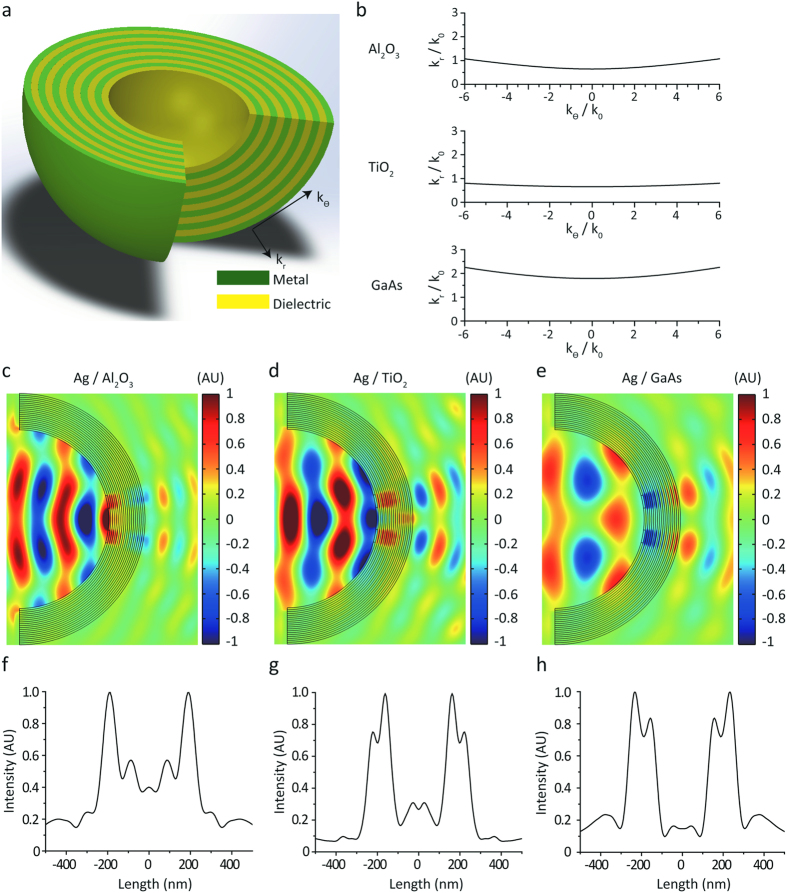
Basic hyperlens structure and simulation results. (**a**) Multilayered spherical hyperlens structure. Metal and dielectric thin films are deposited on a spherical shape of substrate. High-frequency information can propagate along the radial direction without decaying and be captured in the far-field. (**b**) Normalized isofrequency contours of three combinations of materials; Al_2_O_3_, TiO_2_ and GaAs as dielectrics with silver as metal. All combinations satisfy considerably flat hyperbolic dispersion. (**c**–**e**) The normalized magnetic field distribution is shown in 2D simulations. Sub-wavelength waves propagate along the radial direction further to the far-field and are magnified while passing through the hyperlens in each case: (**c**) Ag/Al_2_O_3_, (**d**) Ag/TiO_2_ and (**e**) Ag/GaAs. The normalized power flux density in the cross-section of the hyperlens is shown for (**f**) Ag/Al_2_O_3_, (**g**) Ag/TiO_2_ and (**h**) Ag/GaAs.

Hyperlens is a metamaterial based imaging device whose super-resolving power comes from the hyperbolic dispersion relation in anisotropic media. Figure 1(a) shows the hyperlens structure consisting of a metal and dielectric multilayer. Each layer of a metal and dielectric film is alternately deposited on the spherical shape of the substrate, resulting in the anisotropic characteristic. In the effective medium approximation^29^ describing the macroscopic properties of composite materials, the permittivity values of the multilayer are given by


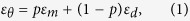



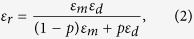


where *p* is the filling ratio of the metal, *ε*_*m*_ and *ε*_*d*_ are the permittivity of the metal and the dielectric, respectively, and *ε*_*r*_ and *ε*_*θ*_ are the permittivities in radial and tangential directions, respectively. The dispersion of the electromagnetic wave is given by


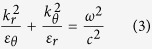


in spherical coordinates for transverse magnetic modes in the medium, where *k*_*r*_ and *k*_*θ*_ are radial and tangential components of wavevector, respectively, ω is the angular frequency, *c* is the speed of light. In a highly anisotropic medium, the permittivities of the radial and tangential components have opposite signs, and the dispersion relation is hyperbolic. When permittivity in the tangential direction is positive and in radial direction is negative (*ε*_*θ*_ > 0 and *ε*_*r*_ < 0), there is no cut-off frequency in the transverse wavevector component, and waves having a large *k*_*θ*_ (which are evanescent waves in isotropic media) can propagate without decaying. Furthermore, the high-frequency information inside the hyperlens is converted to the propagating wave due to the conservation of angular momentum and is sufficiently magnified to be captured by conventional optics systems.

Hyperlenses demonstrated so far have different working frequencies depending on the materials combination, a one-dimensional hyperlens in the UV range using silver/aluminum oxide^15^ and a two-dimensional hyperlens in the visible range using silver/titanium oxide^26^. This shows that, depending on the combination of metal and dielectric materials, a wide range of frequencies can be covered for hyperlens imaging^30^. We used COMSOL Multiphysics 5.1 software and MATLAB R2013a to simulate the performance and to calculate the characteristics of the hyperlens. Figure 1(b) shows the isofrequency contours of three combinations of materials: Al_2_O_3_^31^, TiO_2_^26^, and GaAs^32^. The permittivity of Ag^33^, Al_2_O_3_, TiO_2_, and GaAs are as follows: *ε*_*Ag at* 365 *nm*_ = −2.40 + 0.24*i, ε*_*Ag at* 410 *nm*_ = −4.99 + 0.22*i, ε*_*Ag at* 520 *nm*_ = −11.0.46 + 0.33*i*, 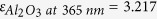, 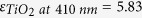, and *ε*_*GaAs at* 520 *nm*_ = 17.418. Each isofrequency contour shows a nearly-flat hyperbolic dispersion shape at the different frequencies. From the UV to visible wavelength range beyond the 500 nm, hyperlenses can have considerably flat hyperbolic dispersion, which means that transverse wavevectors much larger than the cut-off wavevectors can propagate along the radial direction, enabling super-resolution imaging over a wide range of frequencies. Figure 1(c–e) show the normalized magnetic field distribution for each hyperlens design in two-dimensional (2D) COMSOL simulations. The structure in the simulations has both 15 nm thick metal and dielectric multilayer on 2.5 μm diameter half-sphere, where the incoming source is a transverse magnetic plane wave. Two 80 nm diameter holes separated by 160 nm in a chromium (Cr) layer are considered as the sub-diffraction objects. The magnified image of the small objects is formed while passing through the hyperlens and propagating to the far-field. Figure 1(f–h) show the plane cross-section normalized power density flux recorded at the outer surface of the hyperlens corresponding to Fig. 1(c–e), respectively. Fine features of the objects are clearly resolved through the hyperlens. Based on the hyperlens simulations, we selected the Ag/TiO_2_ design as the unit cell for the demonstration of large-scale hyperlens device because of its working wavelength and high resolving power at visible wavelengths. Nevertheless, other designs of the hyperlens shown in Fig. 1 can also be demonstrated by our large-scale fabrication method. The details of the fabrication process are described in the fabrication section.

### Master stamp fabrication

**Figure 2:**
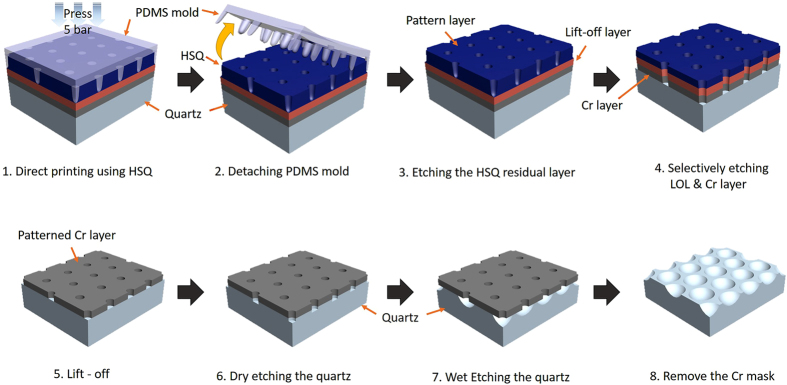
Fabrication procedure of the master stamp for hyperlens.

First of all, we fabricate a quartz master stamp of HAHS patterns. The overall fabrication procedure of the quartz master stamp is shown in Fig. 2. First, a quartz substrate with a Cr layer and a lift-off layer (LOL) are prepared as a mask layer for the selective quartz etching process^34^. A 100 nm thick layer of Cr is deposited on the quartz substrate using a thermal evaporator, and the LOL is coated onto the Cr-layered quartz substrate. For the direct printing process, a soft polymer mold with a hexagonal array of pillar patterns of 1 μm pitch is fabricated using poly dimethylsiloxane (PDMS), which is a polymeric organosilicon compound with a low surface energy^35^. In order to fabricate the hexagonal array of hole patterns on the quartz substrate, hydrogen silsesquioxane (HSQ) is used for its feasibility of solution processing and its simple UV curing process^36^. The hexagonal array of hole patterns is fabricated on the Cr-layered quartz substrate and the LOL by a direct printing technique at 0.5 MPa for 5 minutes. To use the HSQ patterns as a mask layer for the LOL and the Cr layer, a residual layer of the HSQ patterns is etched by a reactive ion etching (RIE) process under the following conditions: CF_4_, 18 sccm; O_2_, 2 sccm; and a 3.33 Pa pressure and 350 W of power applied for 1 minute^37^. Using the HSQ patterns as a mask layer, the lift-off layer is etched selectively using the RIE process under the following conditions: O_2_, 20 sccm, and a 2 Pa pressure with 150 W of power applied for 2 minutes^38^. At the end of the LOL etching process, the Cr layer is etched selectively by an inductively coupled plasma (ICP) process which is set to 1 Pa of pressure, 800 W of ICP power, 50 W of bias power, 10 sccm of O_2_ gas, and 50 sccm of Cl_2_ gas^39^. Next, the HSQ layer and the LOL are removed by a lift-off process. Likewise, the quartz substrate is etched by an ICP process using the Cr layer as a mask. A quartz substrate is vertically etched using the ICP process to fabricate the hemisphere-shaped quartz. Then, the quartz substrate is wet-etched selectively by a buffered oxide etchant (BOE) at a ratio of 1 part BOE to 6 parts deionized water, followed by removal of Cr layer by a Cr etchant solution. Finally, we fabricated the quartz master stamp of the HAHS patterns.

**Figure 3:**
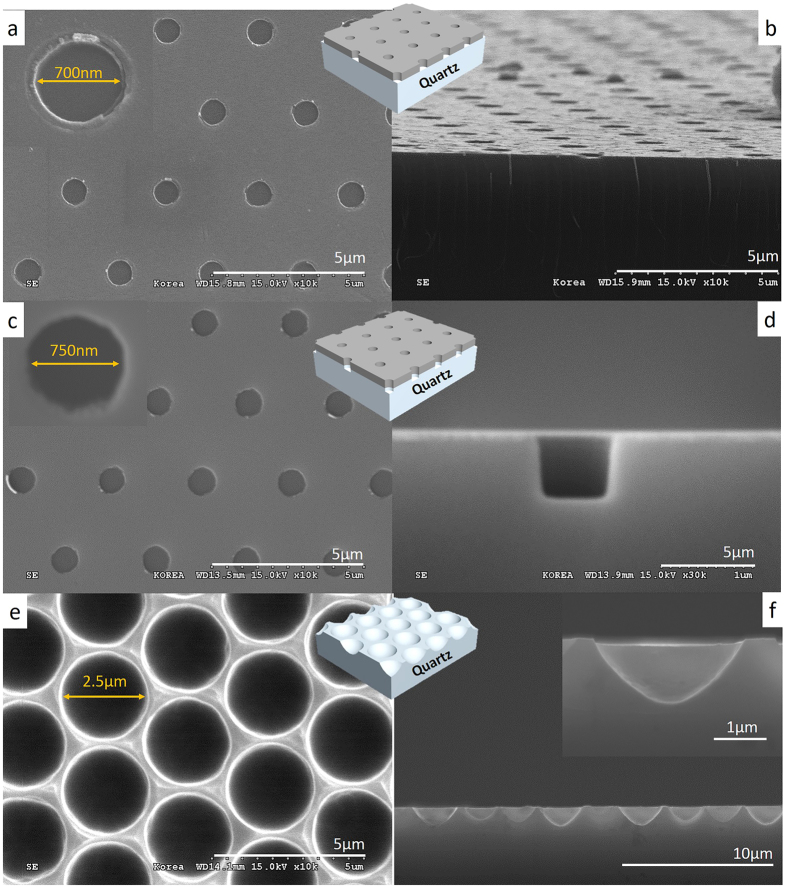
SEM images of the detailed step-by-step master mold fabrication process. (**a**,**b**) SEM images of the top and cross-section views, respectively, after the lift-off process for a hexagonal array of hole patterns with the dimensions of 700 nm diameter and 3 μm pitch at the Cr layer. (**c**,**d**) SEM images of the top and cross-section views, respectively, after the ICP process for a 750 nm diameter and 3 μm pitch hole patterns at the quartz substrate. (**e**,**f**) SEM images of the top and cross-section views after all process is finished. Half-spherical patterns array is well defined.

Figure 3 shows the scanning electron microscopy (SEM) images of each process. Figure 3(a) and (b) are the top and tilted cross-section view, respectively, of the opened Cr mask patterns after the lift-off process. The Cr mask layer is etched by the ICP process, in the shape of hexagonally arrayed hole patterns. The diameter, pitch, and thickness of the hole patterns are 700 nm, 3 μm, and 100 nm, respectively. The hole patterns are made perfectly over the entire area. Figure 3(c) and (d) show the SEM images of the vertically etched process of the quartz. Using the Cr layer as a mask, the quartz substrate is etched selectively. The diameter and the depth of the hole patterns are 750 nm and 450 nm, respectively. Figure 3(e) and (f) are the SEM images after the wet-etching process and the removal of the Cr mask. The quartz is selectively and isotropically wet-etched by using 5:1 buffered oxide etchant (BOE). The master quartz stamp of the HAHS patterns is perfectly fabricated with the large size of 5 cm × 5 cm without any deformation. The diameter of the hemisphere patterns is 2.5 μm and the depth is 1.7 μm, which are used as the base dimensions of the hyperlens device.

### Replicated substrate fabrication

**Figure 4:**
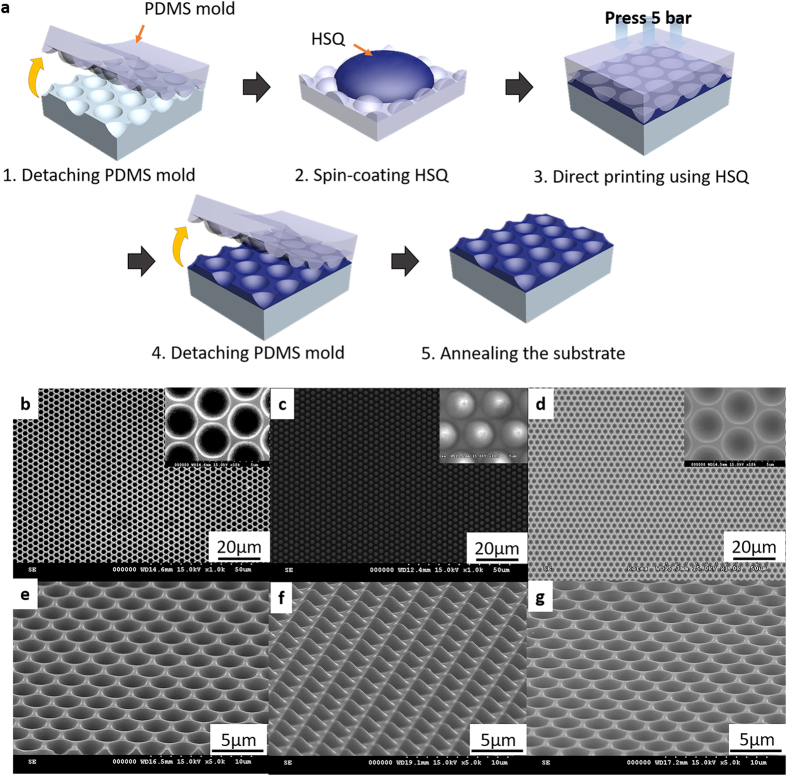
(**a**) Fabrication procedure of the replicated hyperlens substrate. SEM images of (**b**,**c**,**d**) top view and (**e**,**f**,**g**) tilted view for the quartz master mold, the PDMS mold, and the replicated substrate, respectively.

Large-scale HAHS patterned substrate for the hyperlens is simply and easily manufactured by using the quartz master stamp, which is fabricated by deliberate nanoimprinting process. Figure 4(a) illustrates the replication process using a direct printing technique. First, the reverse patterns are manufactured from a master stamp by nanomolding method using PDMS. After a detaching process of the PDMS mold, HSQ solution is spin-coated onto the PDMS mold at 3000 rpm for 30 s^40^. The coated HSQ on the PDMS mold is contacted on the quartz substrate. Then, the PDMS mold is pressed at 0.5 MPa for 5 minutes. After the pressing process, the PDMS mold is detached and the patterns are exposed to UV light for a curing process of 20 minutes. The quartz substrate with the HSQ patterns is annealed using a tube furnace at 500 °C for 1 hour^34^. Figure 4(b) and (e) are the top and tilted view of the SEM images, respectively, of the large-scale quartz master stamp. The HAHS patterns have microconcave structures of 2.5 μm diameter. Figure 4(c) and (f) are the SEM images of the master stamp replicated PDMS mold. The PDMS mold, which has microconvex structures of 2.5 μm diameter and 1.7 μm height, is replicated from the master stamp. Figure 4(d) and (g) are the SEM images of the patterned quartz substrate using the direct printing technique. The pitch and shape of the patterns are exactly the same as those of the master stamp over the whole area, which confirm that the patterns replication is successful.

## Results and Discussions

The curved shape of the hemispherical structure of the HAHS substrate is an important factor in determining the overall shape of hyperlens. Thus, the shape of the replicated patterns must be smooth hemispheres which are exactly same as the shape of the master stamp. In addition, the optical properties of the master stamp and those of the duplicated patterns must be the same. Therefore, the structure shape and components are analyzed using atomic force microscopy (AFM) and x-ray photoelectron spectroscopy (XPS) to compare the replicated patterns and the master stamp.

**Figure 5:**
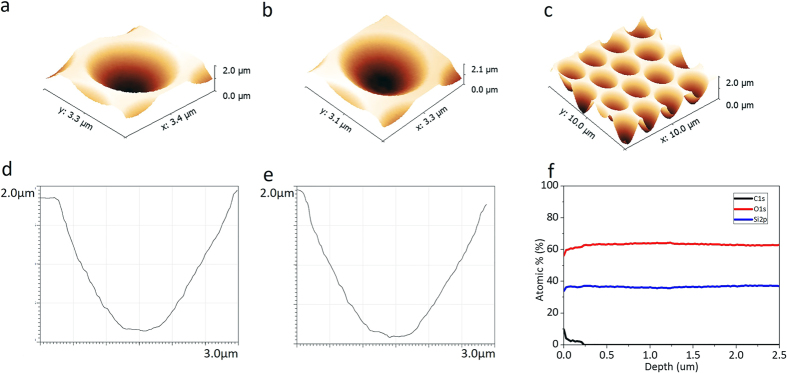
AFM analysis of the master stamp and the replicated substrate for hyperlens array. (**a**–**c**) 3D AFM images of the fabricated patterns on (**a**) master stamp and (**b**,**c**) replicated substrate. The shape has a depth of 1.7 μm and a diameter of 2.5 μm. (**d**–**e**) AFM graph data of the fabricated patterns on the quartz substrate, master stamp (**d**) and replicated substrate (**e**), respectively. (**f**) XPS depth-profiling data of the HAHS-patterned quartz substrate. The carbon signal only exists near the surface region.

Figure 5 displays the AFM and the XPS analysis. The AFM data of the patterns on a replicated substrate, shown in Fig. 5(b) and (e), describes hemispherical structures with 1.7 μm depth and 2.5 μm diameter, which are identical to the AFM data of the master stamp shown in Fig. 5(a) and (d). The AFM data of the replicated patterns array is shown in Fig. 5(c). The XPS analysis graph of the replicated patterns on the quartz substrate (Fig. 5(f)) proves that the composition ratio of the patterned layer and the quartz substrate are equal. As a result, the components and optical properties of the replicated patterns are same as those of the master stamp.

After the HAHS patterned quartz replica substrate is fabricated, nine pairs of 15 nm-thick metal (Ag) and 15 nm-thick dielectric (TiO_2_) are deposited on the substrate using an electron beam evaporator in order to make the final hyperlens device. To prevent the agglomeration of Ag, a deposition process is carried out at low temperature condition, which is controlled by liquid nitrogen below −100 °C. Ag and TiO_2_ layers are deposited alternately with the thicknesses of 15 nm each on a HAHS-patterned substrate, and then a 5 cm × 5 cm size of large-scale hyperlens device is finally fabricated. Experimentally measured permittivity values of TiO_2_ and Ag are 6.61 and −4.19 + 0.42*i* at 410 nm, respectively. They are in general correspondent with the values in the literatures which are used in the numerical simulations.

**Figure 6:**
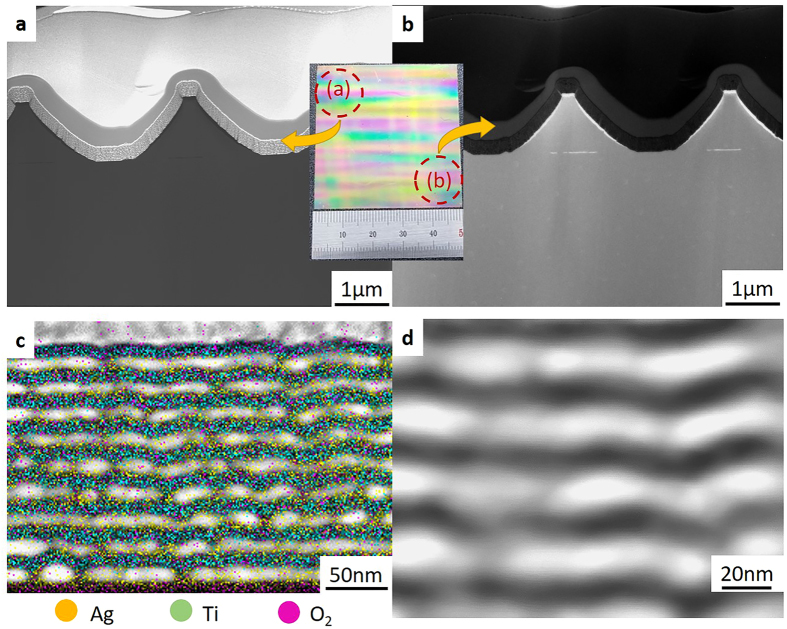
TEM images of the cross-section of a replicated hyperlens. (**a**,**b**) TEM images of both ends of the hyperlens. Both parts have the same shape. (**c**) Ingredients analysis image of the deposited multilayer. (**d**) Zoom-in TEM image of (**c**). The thickness of each layer is 15 nm.

Figure 6 shows the transmission electron microscopy (TEM) images of the hyperlens. Figure 6(a) and (b) show the cross-section views of the TEM images. Those figures reveal that the hyperlens shapes at both ends are identical and multilayer is well defined. Figure 6(c) is a component analysis image of the deposited layers measured by energy-dispersive x-ray spectroscopy. As a result of the component analysis, it is confirmed that each component is detected in accordance with each layer. The TEM image focused on the deposited layer (Fig. 6(d)) verifies that each layer is successfully deposited with the thickness of 15 nm.

**Figure 7:**
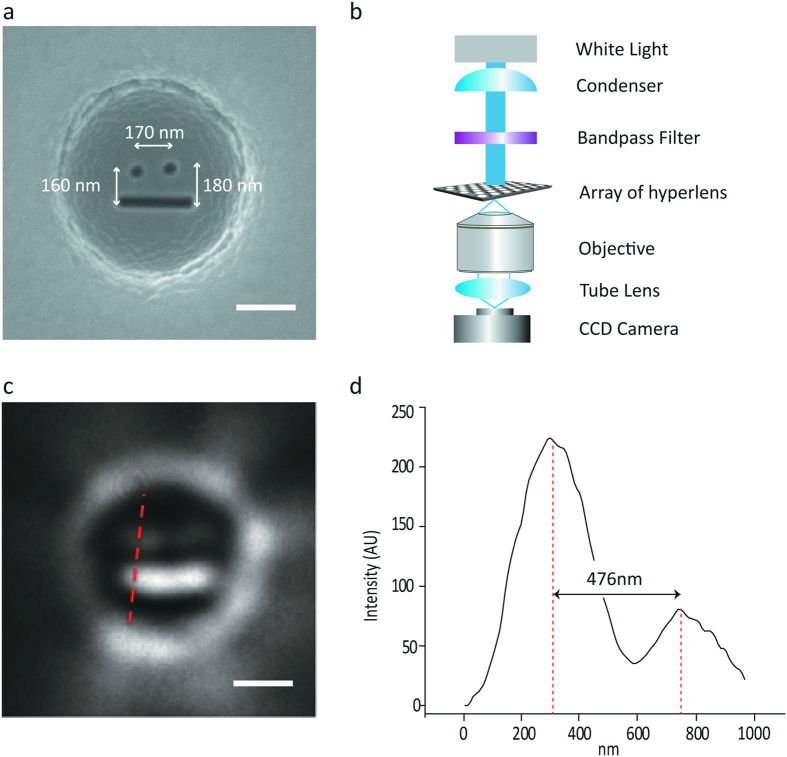
Imaging result with the hyperlens integrated microscope setup. (**a**) SEM image of the sub-diffraction scale objects. 100 nm diameter holes are separated with distance of 170 nm and the distances between each hole and a bar are 160 nm and 180 nm, respectively. (**b**) Optics setup. The hyperlens is illuminated by the selected wavelength of light using bandpass filter and transmitted light is captured by objective lens and CCD camera. (**c**) Far-field optical image after hyperlens. The small object below diffraction limit is clearly resolved by the hyperlens. (**d**) Cross-sectional intensity profile showing 476 nm distance corresponding to 2.97x magnification factor.

Finally, to verify the performance of the hyperlens device, sub-diffractional objects of “smiling face” consisting with two holes and one bar are patterned inside of the hyperlenses. The SEM image of the patterned smiling face is shown in Fig. 7(a). The spacing between the two holes is 170 nm and the holes and bar are separated by 160 nm and 180 nm distance, respectively. The smiling face is imaged by the home-built optical setup shown in Fig. 7(b). An inverted microscope (Axiovert 200, Zeiss) is used as a basic imaging setup body. A white-light mercury lamp (HBO 100, Zeiss) is illuminated through the bandpass filter to select the 410 nm wavelength light. The incident light has no polarization and passes through the hyperlens array sample. The signal that passed through the hyperlens is captured by a 100X oil-immersion objective lens (NA 1.3) and then a sCMOS detector (Zyla 4.2, Andor). The magnified image is shown in Fig. 7(c) and the cross-section intensity profile along the dashed line is plotted in Fig. 7(d). The intensity profile shows a distance of 476 nm in the far-field, corresponding to magnification of 2.97. The result proves the sub-wavelength features magnified in two-dimensions and propagated through the hyperlens are captured by the objective lens and sCMOS camera under the unpolarized illumination. The detailed information about polarization is explained in the Supplementary Information (Figure S3).

In conclusion, we present a scalable fabrication method by adopting a nanoimprinting technique for large-scale hyperlens array device and experimentally demonstrate two-dimensional far-field sub-diffraction imaging with the fabricated device. This solves the main obstacles and limitations of the previous hyperlens devices. Mainly, the difficult and expensive fabrication process is overcome by NIL. In order to easily manufacture a large-scale metamaterial device where hexagonally arrayed hyperlenses are made, we made a quartz master stamp of HAHS patterns and perfectly transferred the HAHS patterns onto the replica substrate. This fabrication method will drastically reduce the cost and enhance the productivity. In addition, the other issue of complicated non-artificial objects positioning in a single hyperlens would be relaxed by providing the high probability of the real objects positioning on the hyperlens array. The proposed imaging system based on the large-scale hyperlens device will provide a simple solution for much more practical far-field and real-time super-resolution imaging which can be widely used in optics, biology, medical science, nanotechnology and other closely related interdisciplinary fields.

## Additional Information

**How to cite this article**: Byun, M. *et al*. Demonstration of nanoimprinted hyperlens array for high-throughput sub-diffraction imaging. *Sci. Rep.* **7**, 46314; doi: 10.1038/srep46314 (2017).

**Publisher's note:** Springer Nature remains neutral with regard to jurisdictional claims in published maps and institutional affiliations.

## Supplementary Material

Supplementary Information
