# Corrigendum: Demonstration of nanoimprinted hyperlens array for high-throughput sub-diffraction imaging

**DOI:** 10.1038/srep46895

**Published:** 2017-08-24

**Authors:** Minseop Byun, Dasol Lee, Minkyung Kim, Yangdoo Kim, Kwan Kim, Jong G. Ok, Junsuk Rho, Heon Lee

Scientific Reports 7: Article number: 46314; 10.1038/srep46314 published online: 04 10 2017; updated: 08 24 2017.

The original version of this Article contained an error in the spelling of the author Minseop Byun, which was incorrectly given as Minsueop Byun.

This has now been corrected in the PDF and HTML versions of the Article.

