# Supplementary material for: Demonstration of nanoimprinted hyperlens array for high-throughput sub-diffraction imaging
Source: Sci Rep. 2017 Apr 10;7:46314. doi: 10.1038/srep46314 (PMC5385565; doi:10.1038/srep46314)
Supplement: Supplementary Information [file srep46314-s1.pdf]

Supplementary Information

Demonstration of nanoimprinted hyperlens array for high-throughput sub-diffraction imaging

Minsueop Byun[1†], Dasol Lee[2†], Minkyung Kim[2], Yangdoo Kim[1], Kwan Kim[1], Jong G. Ok[4], Junsuk Rho[2,3,5*] & Heon Lee[1*]

[1]Department of Materials Science and Engineering, Korea University, Seoul 02842, Republic of Korea

[2]Department of Mechanical Engineering, Pohang University of Science and Technology (POSTECH), Pohang 36763, Republic of Korea

[3]Department of Chemical Engineering, Pohang University of Science and Technology (POSTECH), Pohang 36763, Republic of Korea

[4]Department of Mechanical and Automotive Engineering, Seoul National University of Science and Technology, Seoul 01811, Republic of Korea

[5]National Institute of Nanomaterials Technology (NINT), Pohang 37676, Republic of Korea

[†]These authors contributed equally to this work.

Correspondence and requests for the materials should be addressed to Junsuk Rho (email: jsrho@postech.ac.kr) or Heon Lee (email: heonlee@korea.ac.kr)

1. Confirmation of effective medium approximation with ellipsometry measurement

In this paper, the permittivity values of individual layers are used to calculate the effective permittivity of hyperlens in radial and tangential direction by effective medium approximation (EMA)[1-3]. To confirm the EMA as the effective and accurate technique to apply for hyperbolic metamaterials, metal-dielectric layers are directly measured to obtain the optical properties with ellipsometry. Anisotropic Bruggeman Effective Medium Approaches (ABEMA) is used in ellipsometry of hyperbolic metamaterials, which corresponds to the analytical EMA calculation. Figure S1 shows the n, k values of individual layers of Ag and TiO_2, and one pair of Ag and TiO_2 multilayer.

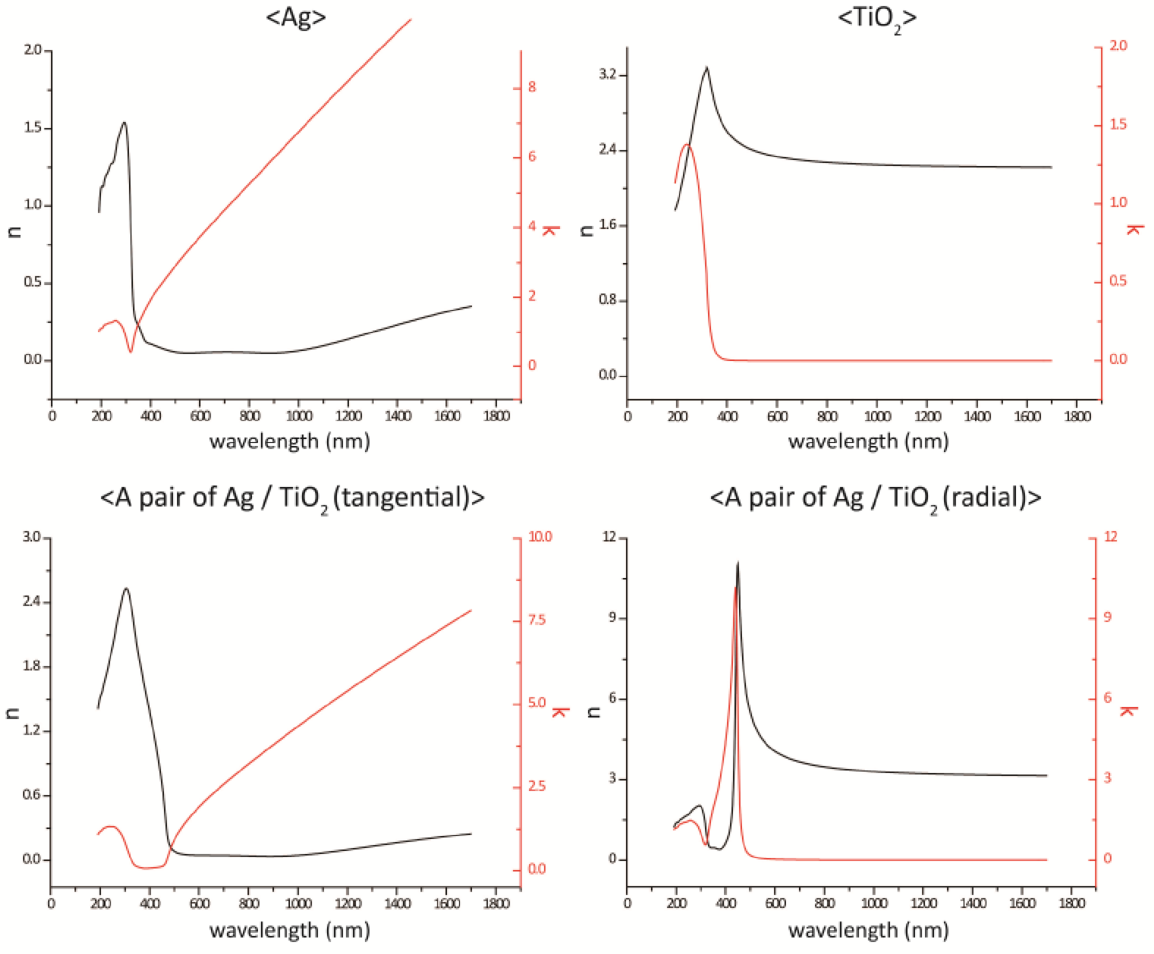

Figure S1. Ellipsometry result (n, k values) of the individual layers (Ag and TiO_2) and a hyperbolic metamaterial (a pair of Ag and TiO_2 multilayer) according to the tangential and radial directions.

The measured permittivity values of individual layer of Ag and TiO_2 are used to calculate the effective permittivity values of multilayered hyperbolic metamaterials, and then compared with the measured permittivity of one pair of Ag and TiO_2 multilayer by ABEMA. The result of comparison between EMA and ABEMA is shown in Figure S2. Although the measurement is done for only a pair of layers due to the limitation of ellipsometry skill and experience, the comparison result in Figure S2 shows the permittivity values of hyperbolic metamaterials in the tangential and radial direction are almost similar in EMA and ABEMA cases. In this manner, EMA method in this paper can be assumed to provide the approximate values of the experimentally measured parameters.

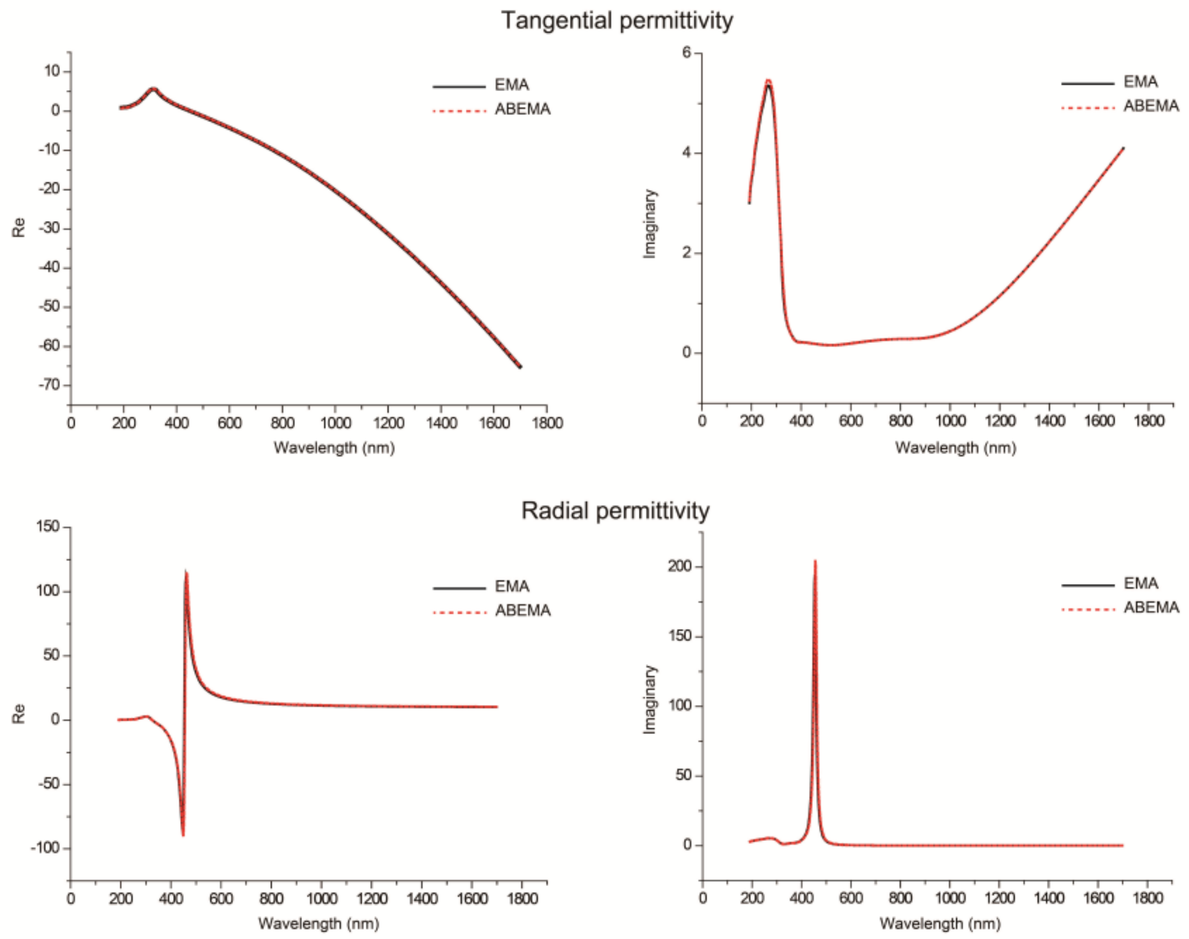

Figure S2. Comparison of the permittivity values of hyperbolic metamaterials between EMA (calculated) and ABEMA (measured). The permittivity values of the tangential and radial directions of EMA and ABEMA are almost similar.

2. Polarization dependency of hyperlens

As the dispersion relations are given by $\frac{k_\perp^2}{\varepsilon_\parallel} + \frac{k_\parallel^2}{\varepsilon_\perp} = k_0^2$ for TM waves and $k_\perp^2 + k_\parallel^2 = \varepsilon_\perp k_0^2$ for TE waves, high transverse wavevector components can be only delivered by TM waves. Therefore, the previous hyperlens[4,5] with cylindrical structure offers one-dimensional super-resolution imaging under linearly polarized light illumination. However, spherical hyperlens[6] overcome this limitation by illuminating the sample with unpolarized light. Since unpolarized illumination provides transverse magnetic components which span the whole two-dimensional reciprocal space, waves containing sub-wavelength features can be propagated in two-dimensions. The experimental comparison of illumination between linearly polarized light and unpolarized light is shown in Figure S3. The linearly polarized light is illuminated to the same smiling face objects in Figure 6(b). The results show that without unpolarized light illumination, 2D sub-wavelength features cannot be resolved in two-dimensions (Figure S3, b-c). Figure S3 (a) proves that under the unpolarized light illumination, spherical hyperlens can perform truly 2D far-field super-resolution imaging.

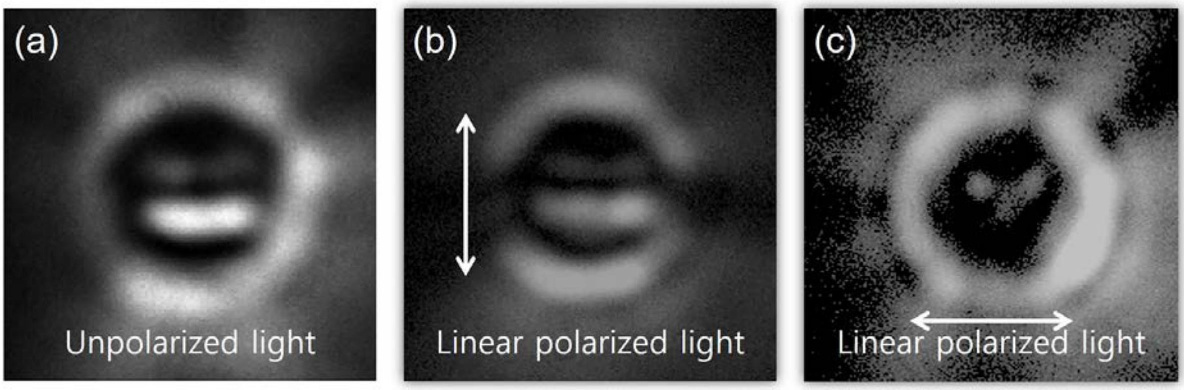

Figure S3. Comparison of the imaging result with the different polarization status. (**a**) Under unpolarized light illumination, sub-wavelength objects consisting of two holes and a bar can be resolved clearly in two-dimensions. (**b**) Under the vertically polarized light illumination, two holes cannot be resolved. (**c**) Under the horizontally polarized light illumination, two holes can be resolved clearly, but the bar cannot be resolved.

References

1. Liu, Y., Bartal, G. & Zhang, X. All-angle negative refraction and imaging in a bulk medium made of metallic nanowires in the visible region. *Opt. Express* **16**, 15439-15448 (2008).
2. Wang, M., Pan, N. Predictions of effective physical properties of complex multiphase materials. *Mat. Sci. and Eng.* **63**, 1 (2008).
3. Stroud, D., Generalized effective-medium approach to the conductivity of an inhomogeneous material. *Phys. Rev. B* **13**, 3368-3373 (1975).
4. Liu, Z., Lee, H., Xiong, Y., Sun, C. & Zhang, X. Far-field optical hyperlens magnifying sub-diffraction-limited objects. *Science* **315**, 1686-1686 (2007).
5. Lee, H., Liu, Z., Xiong, Y., Sun, C. & Zhang, X. Development of optical hyperlens for imaging below the diffraction limit. *Opt. Express* **15**, 15886-15891 (2007).
6. Rho, J. et al. Spherical hyperlens for two-dimensional sub-diffractional imaging at visible frequencies. *Nat. Commun.* **1**, 143 (2010).
